# Predictors of Adherence and Response to Exercise Interventions in Schizophrenia

**DOI:** 10.1192/j.eurpsy.2024.53

**Published:** 2024-08-27

**Authors:** L. Röll

**Affiliations:** Psychiatry and Psychotherapy, LMU Hospital, Munich, Germany

## Abstract

**Abstract:**

Exercise can be considered a feasible and efficient add-on treatment in schizophrenia. However, there remain two major challenges with regard to feasibility and efficiency: First, drop-out rates during exercise programs appear to be relatively high, ranging between 30 to 80 percent. Second, only between 30 and 50 percent of patients clinically respond to exercise interventions. Hence, we aimed to identify factors that predict adherence and response to exercise programs in people with schizophrenia. Based on data from 180 patients with schizophrenia enrolled in the Enhancing Schizophrenia Prevention and Recovery through Innovative Treatments (ESPRIT) C3 study, we examined clinical baseline characteristics that may predict study completion and number of attended trainings (adherence), as well es clinically relevant improvements in symptomatology and functioning (response). We found that only levels of functioning at baseline, but not symptom severity, cognitive functioning, or physical health, predicted adherence. Further, we provide preliminary evidence suggesting that patients with higher cognitive abilities and higher education who performed regular exercise already prior to the study participation were more likely to respond. To conclude, our findings indicate that exercise is particularly helpful for a subgroup of patients characterized by higher levels of functioning, higher cognitive abilities and education, and more pronounced affinity to exercise. Future studies should additionally include environmental, genetic, and neural data to predict adherence and response to exercise.

**Disclosure of Interest:**

None Declared

